# Rapid Volumetric Bioprinting Coupled with Dynamic Perfusion Enhances Human Hepatic Organoid Toxicity Testing

**DOI:** 10.3390/cells15151342

**Published:** 2026-07-27

**Authors:** Yu Tao, Paulina Núñez Bernal, Núria Ginés Rodriguez, Manon Christel Bouwmeester, Tom J. G. Walraven, Dave Wanders, Linda Kock, Nura van Heck, Kerstin Schneeberger-Verjaal, Luc J. W. van der Laan, Bart Spee

**Affiliations:** 1 Department of Clinical Sciences, Faculty of Veterinary Medicine, Regenerative Medicine Center Utrecht, Utrecht University, 3584 CT Utrecht, The Netherlands; y.tao@uu.nl (Y.T.);; 2 Department of Orthopaedics, University Medical Center Utrecht, Utrecht University, 3584 CX Utrecht, The Netherlands; 3 Division of Toxicology, Wageningen University and Research, 6708 WE Wageningen, The Netherlands; 4 LifeTec Group BV, 5611 ZS Eindhoven, The Netherlands; 5 Department of Surgery, Erasmus MC Transplant Institute, University Medical Center Rotterdam, 3015 GD Rotterdam, The Netherlands

**Keywords:** drug-induced liver injury, volumetric bioprinting, perfusion system, hepatic in vitro model, acetaminophen, organoids, cholangiocyte, disease modeling, tissue engineering, hepatotoxicity

## Abstract

**Highlights:**

**What are the main findings?**

- Unlike static cultures, dynamic perfusion of volumetrically bioprinted hepatic organoids promotes functional maturation, as demonstrated by increased expression of key hepatic markers.
- The perfused 3D hepatic constructs are sensitive to extended subtoxic acetaminophen (APAP) exposure, recapitulating key features of continuous drug-induced cellular injury.

**What are the implications of the main findings?**

- Integration of a custom continuous-perfusion bioreactor with volumetric bioprinting provides a feasible route to maintain structurally supported in vitro 3D liver models.
- This dynamic platform has potential for assessing long-term drug-induced liver injury (DILI) and may provide an alternative for pre-clinical hepatotoxicity screening and tissue engineering applications.

**Abstract:**

Drug-induced liver injury (DILI) remains a major cause of acute liver failure and drug withdrawal from the market. Recently developed three-dimensional (3D) hepatic in vitro systems exhibit improved functionality and drug sensitivity compared with conventional two-dimensional cultures. These 3D models range from simple physiologic-like culture systems to advanced bioreactors with dynamic flow to provide sufficient nutrients and consistent drug exposure. However, whether dynamic perfusion improves sensitivity and reproducibility of hepatotoxicity testing remains unclear. Here, we developed a tailor-made perfusion platform to support volumetric bioprinted hepatic constructs for hepatotoxicity testing. The constructs consist of intrahepatic cholangiocyte organoids (ICOs) differentiated towards hepatocyte lineage and embedded in a gelatin methacryloyl bioresin. For toxicity evaluation, the hepatocyte-like ICO constructs were exposed to prolonged subtoxic acetaminophen treatment (10 mM, 7 days). The perfusion system effectively maintained and enhanced hepatocyte differentiation, evidenced by upregulated hepatic markers under perfused conditions compared to static controls. Testing of acetaminophen hepatotoxicity revealed that the perfused constructs displayed elevated cellular injury, with markedly higher liver injury markers relative to controls. Collectively, this study demonstrates the successful application of perfusion-based 3D model culture and highlights its potential as a more physiological platform for hepatotoxicity risk assessment in drug discovery and regenerative medicine.

## 1. Introduction

In Western countries, drug-induced liver injury (DILI) accounts for about 10–52% of acute liver failure [1,2]. DILI accounts for approximately 60% of acute liver failure cases in the United States [3]. Moreover, hepatotoxicity screening is a critical requirement in drug approval procedures, and numerous pharmaceutical compounds have been withdrawn from the market due to hepatotoxic adverse effects [4,5,6]. Thus, there is an urgent need for suitable in vitro liver models for hepatotoxicity screening and continuous research on DILI.

The gold standard for in vitro liver research is primary human hepatocytes (PHH), which are also the most sensitive cell type for drug hepatotoxicity testing [7,8,9]. However, increasing evidence indicates that the biochemical and physiological context of the hepatic cells is essential for drug responsiveness screening in culture [10,11,12]. Cellular metabolic activity and enzyme function provide critical insights into the molecular mechanisms of cytotoxicity and drug efficacy. Cytochrome P450 3A4 (CYP3A4), a predominant phase I enzyme responsible for metabolizing approximately 50% of clinical drugs, generates reactive metabolites that can induce hepatocellular damage [13,14]. Unfortunately, conventional culture methods of PHH fail in maintaining drug-metabolizing enzyme activity as a result of hepatocyte dedifferentiation during prolonged culture and exposure. To overcome the limitations of PHH, stem cell-derived hepatocytes from differentiated induced pluripotent stem cells (iPSCs) or adult tissue-derived liver organoids represent a promising alternative [15]. Both iPSCs and tissue-derived organoids can differentiate into hepatocyte-like and cholangiocyte-like cells under defined growth factor conditions and have been utilized to generate hepatic organoids for drug metabolism studies [16,17,18,19]. Moreover, studies with primary hepatocyte 3D spheroids, such as the large-scale evaluation of 123 compounds by Vorrink et al., demonstrate that 3D models can reveal hepatotoxicity at lower, clinically relevant concentrations, highlighting their sensitivity for drug screening applications [20].

Microfluidic platforms can provide nutrient and oxygen supply to cells during long-term culture, thereby offering more physiologically relevant conditions in vitro, and can achieve physiological exposure to compounds over prolonged periods to study chronic hepatotoxicity [21,22,23,24,25]. In 2015, Nelson et al. applied a perfusion bioreactor for human hepatoblastoma cells to achieve prolonged culture and drug toxicity testing [26]. In 2016, Bhise et al. applied this approach to fabricate a perfusable bioreactor for hepatic spheroids to allow long-term cell culture [27]. Similarly, Tsamandouras et al. designed a flow bioreactor for the stable culture of cryopreserved hepatocytes in 2017 [28]. Toh et al. cultured rat hepatocytes in the microfluidic device and evaluated the hepatotoxicity of drugs. The results indicated that rat hepatocytes cultured in the microfluidic device exhibited toxicity responses [29]. However, the sensitivity of cells in this microfluidic device was not sufficiently high, as the EC50 value of acetaminophen was higher than clinically relevant concentrations. Thus, the application of microfluidic-based organ-on-chip systems for liver toxicity assessment remains under development.

To build a better microfluidic platform, microfabrication techniques are used to create microstructures to mimic the microenvironment where cells function in vivo. Widely used microfabrication techniques can be classified into extrusion-based, inkjet-based, laser-assisted, and light-based bioprinting [30,31]. Volumetric bioprinting (VBP) is a novel and unique contactless light-based bioprinting approach that facilitates the generation of functional cell-laden constructs [32]. This technique relies on the precise delivery of tomographic light projections onto cell-laden photopolymers, resulting in a precise dose distribution that enables the photocrosslinking of large 3D structures in tens of seconds; additionally, the process has been shown to be biocompatible [32,33,34]. Unlike extrusion-based bioprinting techniques, VBP can fabricate entire objects faster than conventional layer-by-layer approaches. This technique protects organoids from shear stress typically sustained during extrusion-based bioprinting, where organoids pass through a nozzle. In a previous study, we successfully applied the VBP technique to shape liver-derived organoids into complex and perfusable architectures, exhibiting high metabolic activity and shape-dependent functionality after bioprinting [35]. While it established a promising in vitro liver platform, focusing on the convergence of organoid technology with VBP, this study did not explore the platform’s potential for drug toxicity assessment screening. Therefore, the aim of the present study is to investigate whether the VBP of perfusable, gelatin methacryloyl (GelMA)-embedded intrahepatic cholangiocyte organoids cultured in a robust dynamic perfusion system can be applied for hepatotoxicity testing. Here, we focused on acetaminophen, the most commonly used analgesic and the most common cause of acute liver injury in the world. Using our novel perfusion system, we assessed metabolic activity and hepatic gene expression during the differentiation period, as well as metabolic activity and metabolic functions under long-term acetaminophen exposure. We show that combining volumetric bioprinting and microfluidics provides a good platform for toxicity testing using human liver-derived organoids.

## 2. Materials and Methods

### 2.1. Organoid Culture, Expansion and Differentiation in Matrigel

Intrahepatic cholangiocyte organoids (ICOs) were cultured in Matrigel™ (Corning, New York, NY, USA) droplets with expansion media (EM) that consisted of Advanced DMEM/F12 (Life Technologies, Carlsbad, CA, USA) supplemented with 1% (*v*/*v*) penicillin-streptomycin (Life Technologies, Bleiswijk, The Netherlands), 1% (*v*/*v*) GlutaMax (Gibco, Dublin, Ireland), 10 mM HEPES (4-(2-hydroxyethyl)-1-piperazineethaanesulfonic acid Gibco, Dublin, Ireland), 2% (*v*/*v*) B27 supplement without vitamin A (Invitrogen, Carlsbad, CA, USA), 1% (*v*/*v*) N2 supplement (Invitrogen, Carlsbad, CA, USA), 10 mM nicotinamide (Sigma-Aldrich, St Louis, MO, USA), 1.25 mM N-acetylcysteine (NAC; Sigma-Aldrich, St Louis, MO, USA), 10% (*v*/*v*) R-spondin-3 conditioned media (Immunoprecise Antibodies, Victoria, BC, Canada), 10 µM forskolin (Sigma-Aldrich, St Louis, MO, USA), 5 µM A83-01 (transforming growth factor beta inhibitor; Tocris Bioscience, Bristol, UK), 50 ng/mL EGF (Invitrogen, Carlsbad, CA, USA), 25 ng/mL HGF (Peprotech, Rocky Hill, NJ, USA), 0.1 µg/mL FGF10 (Peprotech, Rocky Hill, NJ, USA), and 10 nM recombinant human (Leu15)-Gastrin I (Sigma-Aldrich, St Louis, MO, USA). For static culture, expansion media were refreshed every 3 to 4 days, and ICOs were passaged every 7 to 10 days at ratios ranging from 1:2 to 1:4, depending on expansion rate. Cells were kept in a humidified atmosphere of 5% CO_2_ at 37 °C during culture. Organoids were primed for differentiation by adding 25 ng/mL Bone Morphogenic Protein 7 (BMP7; Peprotech, Rocky Hill, NJ, USA) three days prior to the change to differentiation media (DM). DM consisted of the same growth factors as EM, except for R-spondin-3, FGF10, and nicotinamide, and was supplemented with 100 ng/mL FGF19 (Peprotech, Rocky Hill, NJ, USA), 500 nM A83-01 (Tocris Bioscience, Bristol, UK), 10 µM DAPT (Selleckchem, Munich, Germany), 25 ng/mL BMP-7, and 30 µM dexamethasone (Sigma-Aldrich, St Louis, MO, USA). Organoids were differentiated for 10 days prior to acetaminophen (APAP) exposure.

### 2.2. Organoid Expansion in Spinner Flasks

ICOs were isolated from healthy liver biopsies (three independent donors, two female and one male) that were obtained during liver transplantation at the Erasmus Medical Center Rotterdam, The Netherlands, in accordance with the ethical standards of the institutional committee for the use of tissue in research (ethical approval number MEC 2014-060). ICOs were cultured in spinner flasks following a previously published protocol [19]. In short, ICOs were cultured in disposable 125 mL spinner flasks (Corning, New York, NY, USA) to improve expansion. Spinner flasks were inoculated with 2.5 million cells in 25 mL EM, including 10% (*v*/*v*) Matrigel™ (Corning, New York, NY, USA). The spinner flasks were placed on a magnetic stirring plate with the rotation speed set at 85 rpm. Fresh EM and Matrigel™ (Corning) were added every 2–3 days. After 3 weeks, organoids were collected for bioprinting.

### 2.3. Volumetric Bioprinting

Gelatin-methacryloyl was prepared from gelatine derived from porcine skin (Sigma-Aldrich) as previously reported and it was used as a 5% (*w*/*v*) solution in phosphate-buffered saline [35]. The photoinitiator lithium phenyl (2,4,6-trimethylbenzoyl)phosphinate (LAP; Tokyo Chemical Industry, Tokyo, Japan) was added at 0.1% (*w*/*v*) to induce a photocrosslinking reaction. Additionally, the bioink was supplemented with 10% (*v*/*v*) iodixanol for refractive index matching purposes (OptiPrep; StemCell Technologies, Vancouver, BC, Canada). ICOs were resuspended in the bioink at a density of 5 million cells per mL and transferred to a cylindrical borosilicate glass vial. The gel was placed on ice to induce reversible thermal gelation in order to prevent ICO sedimentation during printing. This critical step was specifically implemented to prevent ICO sedimentation, thereby ensuring homogeneous cell distribution and loading consistency across all constructs during the volumetric bioprinting process. Volumetric bioprinting of constructs was performed using a Tomolite printer (Readily 3D, Lausanne, Switzerland). The bioprinter projects tomographic images onto the printing vial at a wavelength of 405 nm. These projections were calculated using the Apparite software (Readily3D, b11409a). Constructs were printed in a Schwarz D structure using a light dose of 250 mJ/cm^2^ (printing time: 20.0 s). After printing, the printing vials were heated to 37 °C to melt the unpolymerized bioink, which was subsequently washed away with pre-warmed phosphate-buffered saline (PBS). Finally, the constructs were post-cured in 0.1% (*w*/*v*) LAP in PBS in a CL-1000 Ultraviolet Crosslinker (UVP, Upland, CA, USA) for 5 min to complete the polymerization of the hydrogel.

### 2.4. Components of Perfusion System

**Figure 1:**
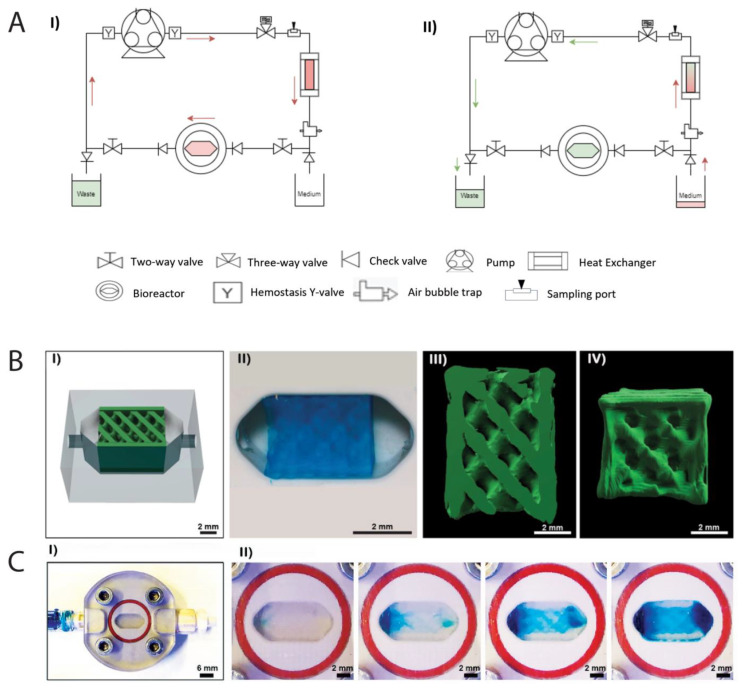
Perfusion setup for volumetrically printed Schwarz D constructs. (**A**) Perfusion system setup with regular flow (**I**) and media refreshment with reverse flow direction (**II**). Red arrows indicate the perfusion of fresh or recirculating culture media, whereas green arrows represent the efflux of spent media into the waste conditioner. (**B**) Volumetrically printed Schwarz D structure for perfusion system. (**I**) Schematic of the inner perfusion chamber with the Schwarz D STL model within it and (**II**) stereomicroscopy image of the GelMA-printed structure (dyed with Alcian Blue staining) in the perfusion chamber. (**III**,**IV**) Light-sheet cross-sectional reconstructions of the printed structure showing an open pore network. (**C**) Perfusability of the volumetrically printed Schwarz D constructs within the perfusion platform. (**I**) Digital image of the assembled perfusion platform with a printed, unstained GelMA construct placed in the chamber. (**II**) Digital images of the construct during perfusion with Alcian Blue dye.

The perfusion system, depicted schematically in Figure 1, is composed of: (1) a polyether ether ketone (PEEK) bioreactor with polycarbonate lid, (2) a roller pump (Ismatec IPC-N, Masterflex, Gelsenkirchen, Germany) with the ability for low speed pumping using Tygon^®^ tubing (Ismatec, LMT-55, 2-stop, 0.19 mm ID; Masterflex) and multiple systems in parallel with a modular setup; (3) a heat exchanger; (4) an air bubble trap (Elveflow, Paris, France); (5) two media containers, one for fresh media and one for waste media. The bioreactor, air bubble trap and heat exchanger are placed inside the incubator.

**Figure 2:**
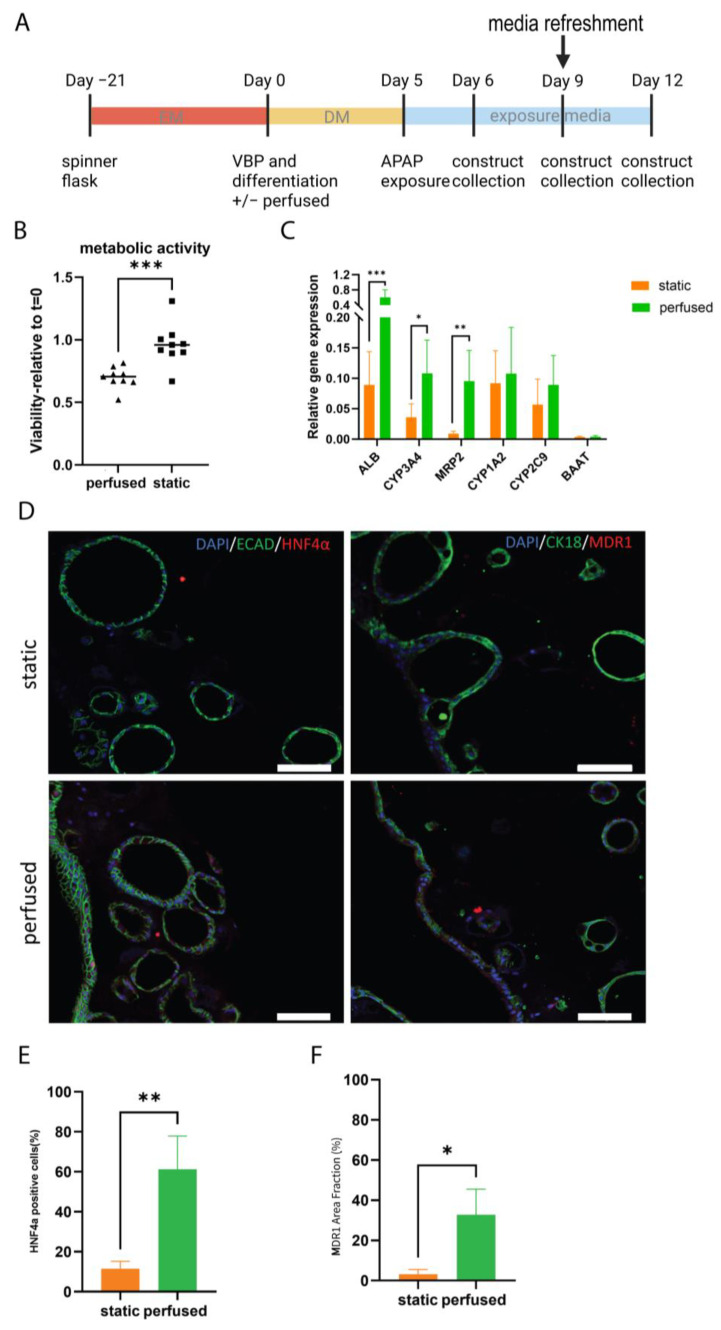
Characterization of ICOs in bioprinted constructs after 5 days of differentiation. (**A**) Schematic of the timeline for the expansion of ICOs, fabrication of bioprinted constructs, and differentiation under perfusion and static conditions, followed by APAP treatment and collection of constructs at different time points. (**B**) Metabolic activity between static and perfused conditions. The data represent *N* = 3 independent donors, with *n* = 3 technical replicates per donor. Triangles and squares represent perfused and static cultures, respectively. (**C**) Relative gene expression of hepatocyte markers in constructs under static and perfused conditions (*N* = 3 independent donors, *n* = 3 technical replicates per donor). Transcriptional levels of albumin (ALB), cytochrome p450 3A4 (CYP3A4), multidrug resistance-associated protein 2 (MRP2), cytochrome p450 1A2 (CYP1A2), cytochrome p450 2C9 (CYP2C9) and amino acid N-acyltransferase (BAAT) were determined by qRT-PCR and normalized to reference genes. * = significant difference (*p* < 0.05), ** = significant difference (*p* < 0.01), *** = significant difference (*p* < 0.001). (**D**) Representative fluorescence images of hepatic constructs. Epithelial marker (ECAD), liver-specific markers (HNF4α and CK18) and drug transporter (MDR1) in static and perfused conditions. Images are representative of *N* = 3 independent donors. Scale bar = 100 μm. (**E**,**F**) Percentage of cells positive and area positive for HNF4α and MDR1. The data represent the means ± S.D. (*N* = 3 independent donors, *n* = 3 images per condition). * = significant difference (*p* < 0.05), ** = significant difference (*p* < 0.01).

The bioreactor consists of two compatible and screwable parts: the PEEK bioreactor with a chamber (dimensions lxwxh 8 × 6 × 6 mm for the rectangular part, total length from inlet to outlet 12.3 mm) for the construct and a polycarbonate lid (Supplementary Figure S1). The bioreactor was designed to be easily milled from PEEK, a high-performance semi-crystalline thermoplastic with limited curvature that can be reproduced with high precision. PEEK has strong mechanical properties with a high resistance to fatigue and wear due to reuse (including sterilization) and flow. The square chamber has triangular ends to equalize the pressure of the fluid before the media enters the construct. For loading, bioprinted constructs were placed in the bioreactor while it was submerged in DMEM media to limit air bubbles in the chamber. The aforementioned one-way valves right before and after the bioreactor allow the system and bioreactor with the construct to be filled separately. The PEEK bioreactor is equipped with inlet and outlet channels with screw threads. Watertightness is achieved by a silicone O-ring at both the inlet, outlet and around the chamber (Figure 2A). Bioreactors were placed in stainless steel holders which were organized in a modular system (Figure 2C) and placed at a 20-degree angle to minimize potential pockets of air in the bioreactor. The total volume of the system was 17–20 mL, including 12–14.5 mL dead volume in the tubing. The media containers can hold up to 120 mL.

Polycarbonate parts (heat exchanger, media and waste containers, lid of bioreactor) were vapor polished with dichloromethane to make them see-through and enable monitoring parameters such as construct integrity and media color during perfusion. Tygon^®^ tubing (1/16 inch inner diameter) was used. An injection port was placed right before the heat exchanger to take media samples during perfusion without disturbing the flow.

### 2.5. Perfusion Experiment

The volumetric bioprinted organoid-laden constructs were transferred to a 24-well plate with 2 mL of DM. Constructs were transferred to a sterile bioreactor chamber and perfused with 17–20 mL of DM at a flow rate of 50 µL/min (*N* = 3, *n* = 3). The flow rate was determined based on the previous literature [36,37,38,39,40,41,42]. Additional constructs were transferred to T25 culture flasks and suspended in 17–20 mL of DM as a static group (*N* = 3, *n* = 3). All constructs were incubated at 5% CO_2_ and 37 °C for 5 days. Cell metabolic activity was quantified using the Alamar Blue assay, as detailed below, at the start of perfusion (day 0) and on day 5. The bioreactor and flow perfusion system were designed by LifeTec Group (Eindhoven, The Netherlands) and Utrecht University to provide a continuous flow to the bioprinted hepatic constructs. The flow perfusion system is designed in such a way that media can be refreshed without the need to detach the system. Reversing the flow direction enables media to be refreshed in the perfusion system; as media are pumped from the fresh media container towards the waste media container, the bioreactor is excluded from this circulation by the use of one-way valves (Figure 1C). The media refreshment procedure is performed on day 4 of acetaminophen exposure.

### 2.6. Acetaminophen Exposure

Acetaminophen (APAP; CAS 103-90-2, Sigma-Aldrich) was dissolved in differentiation media (DM) with DMSO as described above, but without the addition of Glutamax, NAC, and B27. On day 5 of differentiation, constructs were exposed to 10 mM APAP for a total of 7 days (*N* = 2, *n* = 3), with media refreshed every 3 days. At the same time points, media were collected for analysis of hepatocellular injury markers. Metabolic activity was measured with Alamar Blue on days 1, 4, and 7 of acetaminophen exposure.

### 2.7. Alamar Blue Assay

Metabolic activity of organoids in constructs was measured using Alamar Blue Cell Viability Reagent (Invitrogen) in order to quantify metabolic activity. Briefly, the Alamar Blue Cell Viability Reagent was diluted 1:10 in Advanced DMEM/F12 without phenol red (Life Technologies), sterilized through a 0.22 μm filter and pre-warmed to 37 °C. Cells were incubated for two to four hours at 37 °C. Absorbance was measured after incubation at ex/em 544/570 nm using a spectrophotometer (Fluoroskan Ascent FL, Thermo Fisher Scientific, Waltham, MA, USA).

### 2.8. Live/Dead Staining

Cell viability of constructs under APAP exposure was assessed by Live/dead Viability/Cytotoxicity Kit for mammalian cells (ThermoFisher, Catalog number: L3224). Samples were incubated with fluorescent dyes to detect live (Calcein-AM) and dead (Ethidium homodimer-1) cells. Samples were imaged using confocal laser scanning microscopy (SP8, Leica Microsystems, Amsterdam, The Netherlands).

### 2.9. RNA Isolation and RT-qPCR

RNA was isolated from bioprinted constructs using the RNeasy mini kit (Qiagen, Hilden, Germany) according to the manufacturer’s instructions. The constructs were treated using the Cell Collect G kit (LR0200000010, Cellink, Gothenburg, Sweden), which contained collagenase to degrade the hydrogel before RNA isolation. RNA isolation from Matrigel droplets was followed by the RNA extraction protocol of the RNeasy micro kit. RNA concentrations were determined by the DS-11 spectrophotometer (DeNovix, Wilmington, DE, USA). cDNA synthesis was performed using the iScript cDNA synthesis kit (Bio-Rad, Veenendaal, The Netherlands). Relative gene expressions of interest (listed in Supplementary Table S1) were measured using RT-qPCR in a CFX-384 (Bio-Rad). Normalization was carried out using reference genes Ribosomal Protein S5 (RPS5) and Ribosomal Protein L13A (RPL13A).

### 2.10. HE Staining and Immunofluorescence Analysis

For hematoxylin and eosin (H&E) staining, constructs (three independent donors, two female and one male) were fixed for 15 min in 4% paraformaldehyde (PFA) at room temperature, dehydrated, cleared and embedded in paraffin as usual. The paraffin slides were stained using a Leica automatic stainer.

For immunofluorescence staining, constructs were treated by Cell Collect G kit which contained collagenase to degrade hydrogel before fixation. Organoids (three independent donors, two female and one male) were fixed for 15 min in 4% PFA at room temperature and embedded in paraffin. The organoids were incubated overnight with primary antibodies (listed in Supplementary Table S2) at 4 °C, washed three times with PBS and then incubated with secondary antibodies for 1 h at room temperature in the dark. Finally, nuclei were stained with 0.5 μg/mL DAPI (Sigma-Aldrich, St Louis, MO, USA) for 10 min and washed with PBS. Images were captured using confocal laser scanning microscopy (SP8, Leica Microsystems, Amsterdam, The Netherlands).

### 2.11. Immunofluorescence Quantification

Immunofluorescence images were analyzed with Fiji [43]. The total cell number was determined by counting DAPI-stained nuclei for hepatocyte nuclear factor-4α (HNF4α). The percentage of positive cells was calculated as the ratio of marker-positive to total DAPI-positive cells. The expression of multidrug resistance 1 (MDR1) was quantified by the positive area fraction. Z-stack confocal images were processed to maximum intensity projections using Fiji (ImageJ version 1.51h; National Institutes of Health, Bethesda, MD, USA). Each organoid area was defined by the DAPI counterstain. Consistent intensity thresholds were used to isolate specific MDR1 fluorescent signals. The positive area fraction was determined by dividing the thresholded transporter-positive area by the total organoid area.

### 2.12. Statistical Analysis

Statistical analysis was performed using GraphPad Prism (version 10.4.2). The qRT-PCR results in Figure 2C were analyzed using an independent samples *t*-test for intergroup comparisons. Statistically significant differences were set up when * *p* < 0.05.

## 3. Results

### 3.1. Establishing a Perfusion System for the Dynamic Culture of Volumetrically Printed Structures

The perfusion system elements were connected and incubated at 37 °C in 5% CO_2_ (Figure 1A(I) and Figure S1). Media refreshment was performed by reversing the pump flow direction, with fresh and waste media containers connected as shown (Figure 1A(II)). Building on previous results showing that mathematically derived structures provide important architectural features that enhance metabolite exchange in VBP-printed ICOs [35], GelMA constructs with an optimized Schwarz D structure were fabricated via volumetric bioprinting (Figure 1B). The printed structure was specifically designed to fit within the developed perfusion chamber (prism-shaped, 6 × 6 × 8 mm H × W × L; Figure 1B(I,II)). Light-sheet 3D reconstructions revealed that the internal structure of the gyroid comprised an open and interconnected pore network (Figure 1B(III,IV)). A thin outer wall was added to the gyroid network to constrain the flow to the inner pores of the structure and reduce flow outside the pore network, since the construct was not directly connected to the printed structure. To demonstrate the perfusability of the printed constructs, the printed GelMA structures were placed in the perfusion platform and perfused with Alcian Blue dye to visualize flow through the pore network. Results showed the gradual perfusion of the entirety of the printed structure, with minimal fluid accumulation outside the printed structure thanks to the outer wall and tight fit of the print within the perfusion chamber (Figure 1C). Having established an integrated platform allowing for continuous perfusion of volumetrically printed hydrogel structures, we proceeded to employ this platform for the differentiation and toxicity evaluation of printed liver organoids.

### 3.2. Assessment of Volumetrically Bioprinted Liver Organoid Maturation Under Dynamic Perfusion

A schematic representation detailing the integration of volumetric bioprinting and dynamic perfusion culture for the generation and hepatotoxicity testing of printed liver organoids. (Figure 2A). Constructs were cultured either under perfusion (50 µL/mL differentiation media for 5 days) or in static conditions. After 5 days of differentiation, constructs from both conditions were collected and analyzed. Metabolic activity was determined by the Alamar Blue assay. This overall metabolic activity was significantly higher in the static group compared to perfused constructs (*p* < 0.001) (Figure 2B). One explanation is that the effective cell number is reduced due to fluid-induced shear stress that partially washes out loosely attached cells from the hydrogel matrix, rather than a decrease in actual hepatocyte function, since not all cells initially loaded into the vial are captured in the VBP. It is also worth mentioning that the organoids shift to functional differentiation when transferred to differentiation media, and not to cellular expansion. Gene expression analysis, however, revealed significant upregulation of hepatic maturation markers in the perfused group compared to static controls. Specifically, albumin (ALB), multidrug resistance-associated protein 2 (MRP2), and cytochrome P450 3A4 (CYP3A4) were significantly higher in perfused constructs (*p* < 0.001, *p* < 0.01 and *p* < 0.05, respectively) (Figure 2C). Additional hepatic markers, including CYP1A2 and CYP2C9 (both involved in acetaminophen metabolism) and bile acid–CoA thioesterase (BAAT), were also examined to further characterize functional maturation but were not significantly different between groups.

Immunofluorescence analysis confirmed epithelial (E-cadherin, E-CAD) and cytoskeletal (cytokeratin 18, CK18) expression under both conditions. Notably, the hepatocyte marker (hepatocyte nuclear factor-4 alpha, HNF4α) showed a stronger immunofluorescent signal in perfused constructs, consistent with enhanced hepatic differentiation. Moreover, polarization of the organoids appeared improved in perfused cultures, as indicated by increased expression of multidrug resistance 1 (MDR1) (Figure 2D). Quantitative immunofluorescence analysis further confirmed that dynamic perfusion significantly enhanced both hepatic differentiation and cellular polarization, evidenced by a notably higher percentage of cells expressing HNF4α and MDR1 compared to static controls (*p* < 0.01 and *p* < 0.05) (Figure 2E,F).

Taken together, these results indicate that while metabolic activity was higher under static conditions, perfusion culture promoted hepatic maturation of ICO-derived constructs at the transcriptional and structural level.

### 3.3. Acetaminophen-Induced Hepatotoxicity During Long-Term Exposure

**Figure 3:**
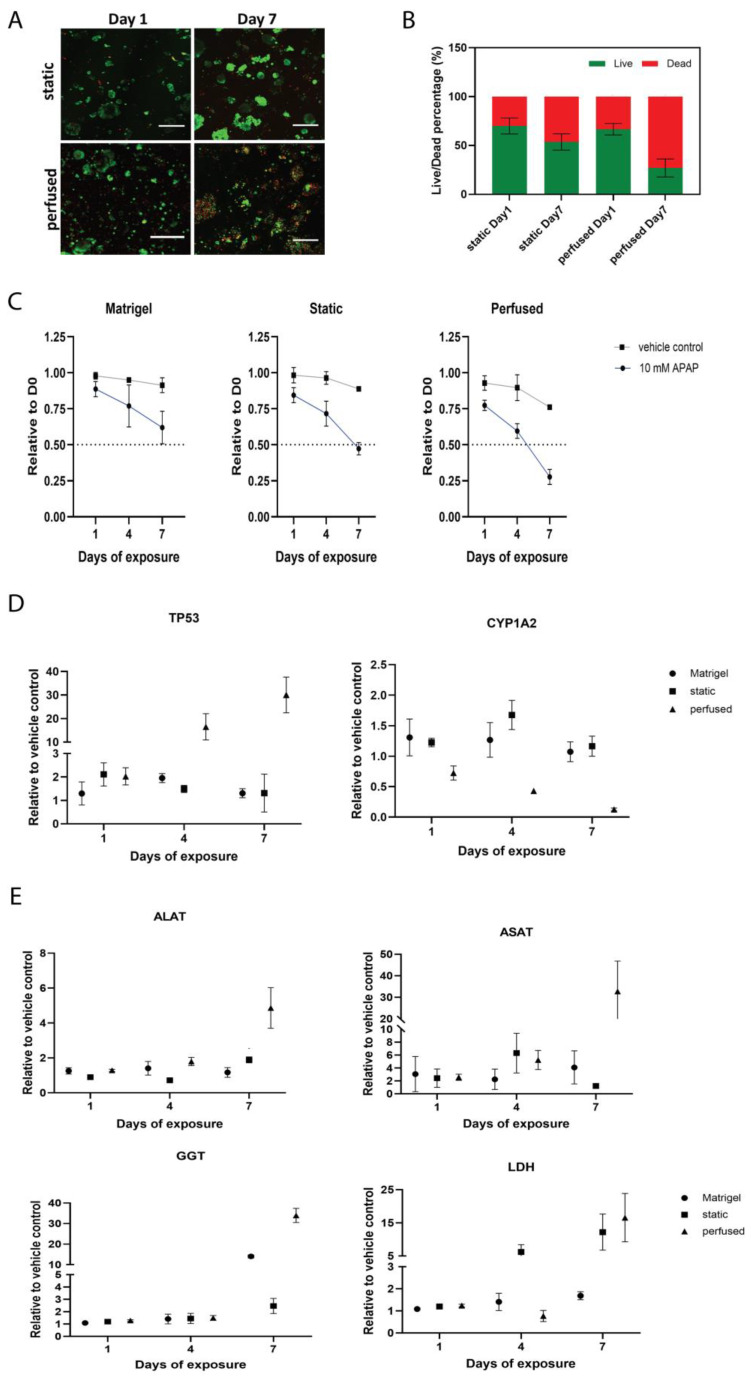
Evaluation of low-dose repeated long-term APAP-induced hepatotoxicity in bioprinted hepatic constructs. (**A**) Live/dead staining was performed to visualize metabolic activity during acetaminophen exposure (10 mM). Green indicates live cells and red indicates dead cells. Scale bar = 25 μm. (**B**) Quantification of live/dead distribution of live/dead staining after 1 day and 7 days of acetaminophen exposure. Data represent *N* = 2 independent donors, with *n* = 3 technical replicates per donor. (**C**) Metabolic activity level in Matrigel, static and perfused conditions during APAP exposure and normalized to vehicle control (0.5% *v*/*v* DMSO). The horizontal dotted line indicates a relative value of 0.5 compared to day 0. Vehicle control and 10 mM APAP treatment are denoted by squares and circles, respectively. (*N* = 2 independent donors, *n* = 3 technical replicates per donor). (**D**) Relative gene expression of hepatic damage marker and the enzyme involved in acetaminophen metabolism in constructs under Matrigel, static and perfused conditions. TP53 and cytochrome P450 1A2 (CYP1A2) were determined by qRT-PCR and normalized to vehicle control. Specific culture conditions are denoted by distinct symbols: Matrigel (circles), static (squares), and perfused (triangles). (*N* = 2 independent donors, *n* = 3 technical replicates per donor). (**E**) Liver transaminase levels were measured in media under Matrigel, static and perfused conditions and normalized to untreated vehicle control. Specific culture conditions are denoted by distinct symbols: Matrigel (circles), static (squares), and perfused (triangles). (*N* = 2 independent donors, *n* = 3 technical replicates per donor).

After 5 days of differentiation, constructs exhibited hepatic functionality, which is essential for drug screening applications. To establish an in vitro model of APAP-induced liver injury, constructs were exposed to 10 mM APAP for up to 7 days. Samples were collected after 1, 4 and 7 days of exposure. Fluorescence-based viability revealed pronounced cell death after 7 days of APAP exposure, indicating cytotoxic stress (Figure 3A,B). Cellular metabolic activity was assessed in Matrigel and in bioprinted constructs cultured statically and under dynamic perfusion. As shown in Figure 3C, APAP exposure led in all conditions to a progressive decrease in metabolic activity compared to baseline activity. In Matrigel cultures, activity declined to ~50% after 7 days, whereas static and perfused VBP cultures showed marked reductions already after 1 day. Remarkably, after 7 days, perfused constructs retained only ~25% of their baseline metabolic activity, compared to ~50% in Matrigel and static conditions (Figure 3B,C). To probe stress responses, gene expression of tumor protein p53 (TP53) and its downstream target cyclin-dependent kinase inhibitor 1A (CDKN1A) was examined. TP53 gene expression exhibited a robust upward trend in perfused constructs compared to Matrigel and static conditions (Figure 3D). In contrast, CDKN1A expression remained comparable across all culture conditions (Figure S4).

To evaluate APAP metabolism, CYP1A2 and 3A4 expression were assessed. CYP1A2 was substantially downregulated under perfusion, whereas CYP3A4 expression remained stable and comparable among all conditions relative to vehicle controls (Figure 3C and Figure S4). Release of liver injury enzymes confirmed higher sensitivity under perfusion. As shown in Figure 3E, after 7 days of APAP exposure, alanine aminotransferase (ALAT), aspartate transaminase (ASAT) and gamma-glutamyl transferase (GGT) levels were markedly higher in perfused constructs compared to Matrigel and static VBP cultures. For lactate dehydrogenase (LDH) levels at day 7, both static and perfused constructs were higher compared to Matrigel. ALAT levels were already notably elevated in perfused constructs compared to Matrigel and static conditions after 7 days (Figure 3E). Collectively, these results demonstrate that volumetrically printed ICOs maintained under long-term perfusion exhibit increased sensitivity to acetaminophen toxicity, thereby improving the predictive capacity of toxicity testing in human hepatic organoids.

## 4. Discussion

Here, we developed an advanced perfusable platform for bioprinted human ICOs and showed its utility for acetaminophen-induced hepatotoxicity assessment. We applied the contactless, high-speed VBP technique, a light-based technology, to fabricate layerless 3D biological constructs within 20 s. This is a custom-designed perfusion bioreactor system that introduced a continuous nutrient supply and mild shear stresses to engineered tissues, promoting tissue maturation [44,45]. Our results showed that hepatic functionality was significantly enhanced in constructs cultured within the bioreactor system compared to those cultured under static conditions. Subsequently, the chronic APAP-induced hepatoxicity evaluation model using liver organoids was established in the perfusion platform.

Hepatic differentiated ICOs are powerful in vitro models for toxicity studies and exploring personalized therapy due to their patient-derived origin [46,47,48]. ICOs can be maintained in long-term culture for expansion and differentiation into functional hepatocyte-like cells (HLCs) in vitro. Currently, Matrigel, which is derived from Engelbreth-Holm–Swarm (EHS) mouse sarcoma, is the most widely used hydrogel matrix for ICO culture, serving as a biological scaffold [49,50]. However, ICOs cultured in Matrigel exhibited progressive loss of functional marker expression, which justifies the use of other scaffolds like GelMA [51]. Furthermore, the use of this very soft material prevents the formation of complex architectures that enable dynamic culture processes crucial to introducing native-like features [52,53]. In this study, we look towards bioprintable materials to encapsulate and mature ICOs in more stable and perfusable systems.

Bioprinting has emerged as a promising technique in regenerative medicine due to its precise spatial control over cell placement and tissue architecture. Several different bioprinting techniques have been used to mimic liver-like tissues to date. Biomedical research teams have devoted significant efforts in recent years to developing cell-compatible bioinks for use with a wide variety of bioprinting strategies, such as drop-on-demand inkjet bioprinting, extrusion-based bioprinting techniques, or light-based bioprinting approaches [54]. However, these conventional layer-by-layer approaches require long printing times to produce large prints like those shown in this study, and pose some limitations on the achievable designs. Furthermore, nozzle-based approaches expose cells to shear stresses, which can compromise viability and alter bioink properties, particularly when working with large, multicellular building blocks like the ICOs [55,56]. In contrast, VBP offers an optimal, contactless approach and allows high-speed fabrication of complex constructs while maintaining precise geometric control [32,33,34,57,58]. In our custom PEEK bioreactor, a Schwarz D triply periodic minimal surface (TPMS) architecture was employed to maximize mass transport [35]. For conventional bulk hydrogels, mass transfer is typically limited [59]; the TPMS design has interconnected open channels that provide the large surface area-to-volume ratio required for dynamic perfusion [60]. The thickness of the lattice wall was limited to 150–200 µm so that the diffusion distances were within physiological limits [61]. The structural control served a dual purpose; it ensured a uniform distribution of nutrients and a homogeneous exposure to APAP. This specific geometrical design avoided the central necrosis characteristic of larger constructs and induced the functional maturation of the organoids, providing a reliable platform for pharmacological testing [62]. To perfuse the printed structures, we employed a robust bioreactor platform manufactured from PEEK. PEEK was selected based on its exceptional chemical resistance, mechanical strength, and biocompatibility, making it well-suited for long-term cultures and exposure to diverse aqueous media [63,64,65]. The system’s modular architecture enables precise control over flow dynamics, shear stress, and nutrient exchange, which are critical parameters in recapitulating physiological conditions in vitro. In contrast to conventional materials such as PDMS-based systems, PEEK provides enhanced durability and autoclave compatibility, enabling repeated use without compromising structural integrity or introducing contamination to the culture system [66].

The dynamic culture in the bioreactor maintained a stable metabolic activity of the VBP liver-like units for at least 5 days. The static cells had a higher overall metabolic activity in Alamar Blue assays, but the initial decrease in metabolic activity under perfusion was expected. The continuous fluid shear stress washes away loosely embedded peripheral cells from the hydrogel matrix inevitably [67].

Within this setup, the organoids themselves serve as biological mechanosensors [68]. The combination of sustained viability and upregulated hepatic markers (ALB, CYP3A4, and MRP2) provides strong evidence that the fluidic environment was not physically destructive. Any excessive shear stress would have inevitably triggered necrotic or dedifferentiation responses, which were conspicuously absent [29,69]. To ensure fluid actually passed through the porous network, the Schwarz D constructs were designed to match the exact dimensions of the PEEK chambers (8 × 6 × 6 mm) and featured a continuous thin outer wall. Following bioprinting and incubation, the natural volumetric swelling of the GelMA hydrogel created a tight interference fit [70]. This precise fit, combined with the structural barrier of the outer wall, effectively minimizes fluid bypass at the tissue boundaries, as corroborated by the temporal flow dynamics in our Alcian Blue perfusion assay (Figure 1C). Importantly, the reliable assembly of these parts underscores the exceptional print-to-print consistency of our volumetric bioprinting process [35,42].

While mechanical cues are vital, the chemical dimension of convective mass transport—specifically oxygen delivery—is equally decisive for hepatic maturation. To rule out the diffusion limitations and hypoxic core formation that frequently plague static 3D cultures, we deliberately scaled our perfusion parameters against the metabolic bulk of the construct [71]. The initial seeding density of the bioink was approximately 5 × 10^6^ cells/mL. Given the maximum geometric volume of the PEEK bioreactor chamber is 288 μL (8 × 6 × 6 mm). Even assuming the chamber was completely filled with the bioink at our seeding density of 5 × 10^6^ cells/mL, the theoretical maximum cell number would not exceed 1.44 × 10^6^ cells. While mature primary human hepatocytes can exhibit peak oxygen consumption rates of 1 fmol/cell/s [72], stem cell- or organoid-derived hepatic constructs in 3D matrices typically exhibit more conservative metabolic profiles [73]. Specifically, within dynamic microfluidic environments, such immature models (e.g., HepG2/C3A or iPSC-derived hepatocytes) rely predominantly on glycolysis and demonstrate significantly lower baseline respiration [74]. Based on standard extracellular flux analyses (Seahorse XF) of such metabolically comparable cell lines, typical basal OCRs fall within the range of 10–20 pmol/min per 10^4^ cells [75]. So the upper bound of oxygen demand is approximately 1.73 nmol/min. With a steady applied flow rate of 50 µL/min (providing a constant influx of ~10 nmol/min based on ~0.2 mM dissolved O_2_), the continuous perfusion ensures that the oxygen supply adequately meets the cellular demand. Thus, it is very likely that the observed functional gains are a synergistic response: the convective flow secures the strict normoxia needed to fuel energy-intensive CYP450 metabolism [76], while the concurrent shear stress provides the mechanotransductive signals needed for epithelial polarization [69].

As a proof-of-concept, we evaluated APAP toxicity in our VBP-perfusion system. Based on previous research, the APAP exposure concentration was selected as 10 mM, which is lower than the EC50 concentration of ICOs, to evaluate sub-hepatotoxicity over 7 days of exposure [77]. TP53 plays a key role in oxidative stress responses during acetaminophen-induced liver injury and has been shown to exhibit protective effects in murine models [78]. Fan et al. reported increased TP53 gene expression during the acute injury phase, accompanied by increased ASAT and ALAT levels within 12–24 h after toxic APAP dosing in mice [79]. In contrast, in our system, TP53 upregulation and ASAT and ALAT release were observed after 4 days, with markedly higher levels in the perfused condition compared to Matrigel and static conditions. This delayed response likely reflects the lower, sub-cytotoxic APAP dose applied, which was insufficient to induce acute oxidative stress within 24 h. Importantly, this distinct temporal dynamic perfectly recapitulates the chronological progression of chronic APAP toxicity [80,81]. Figure 3C shows an initial retention of metabolic activity, but the early decrease in metabolic activity indicates initial mitochondrial dysfunction induced by NAPQI [82,83]. This resulted in a delayed massive release of transaminases (Figure 3E) and a sharp upregulation of TP53 (Figure 3D) until day 7, when the cumulative toxic insult finally overcame cellular defenses and resulted in irreversible necrosis [84]. Furthermore, GGT, a biomarker associated with cholestatic or biliary injury [1], was elevated in both perfused and Matrigel conditions compared to static after 7 days of APAP exposure. This may indicate the presence of residual cholangiocyte-like cells within the hepatic-like organoids, which also respond to APAP with GGT release [85]. The extensive necrosis and the intense release of ALAT, ASAT and GGT confirm our perfused tissues bioactivated APAP successfully [86]. In traditional models and in vivo, this toxicity is localized to the perivenous zone. Hypoxia supports high CYP2E1 activity there naturally [87]. Our perfusion system continuously delivers oxygen, thus entirely bypassing this classical hypoxic niche. The absence of hypoxia along with mechanical flow creates a setting that favors CYP3A4 expression instead [88].

Since CYP3A4 is very effective in converting APAP to NAPQI, the toxic cascade still occurs [89]. The flow conditions kept high and stable levels of CYP3A4 over 7 days, feeding continuous NAPQI accumulation. This dynamic is not a flaw but a unique advantage: it models APAP toxicity only under normoxic conditions. The platform allows the researchers to study the CYP3A4-mediated liver injuries that are often masked in the standard CYP2E1-centric models. Hence, the decrease in CYP1A2 during the perfusion is not the cause of the decrease in bioactivation but a downstream pathological consequence of the injury induced by NAPQI. The quick decrease in certain enzyme transcripts is consistent with the previously reported acute APAP toxicity profiles in HepaRG cells, as advancing necrosis disrupts the transcriptional machinery [90]. Collectively, these results underscore the perfusion system’s potential as a complementary tool in drug development and regenerative medicine. This study was conducted with ICOs derived from only two donors. Expanding the donor pool and including samples across diverse age groups would improve generalizability. A recent study identified age as an independent risk factor for drug-induced liver injury in patients receiving high-dose APAP [91]. Despite robust functional information, the current assay presents two major practical limitations. First, the absence of 3D visual confirmation implies that we cannot directly chart deep-scaffold cellular distribution. To solve this, we need in situ tomography to rule out internal necrosis. Second, it is difficult to follow the metabolic shifts in the PEEK setup due to the lack of real-time dissolved oxygen (DO) sensors. Designs in the future will have to incorporate inline sensing to unambiguously distinguish the effects of oxygen availability from mechanical shear stress.

Beyond the liver, this VBP-perfusion approach is readily adaptable to other high-flow organs. In particular, for the kidney, the replication of tubular flow and shear stress is crucial for the development of robust nephrotoxicity models [92,93]. Integration of these particular organ units into larger multi-organ platforms will substantially improve our capacity to investigate drug pharmacokinetics and systemic crosstalk [94].

## 5. Conclusions

This study introduces a perfusion platform fabricated from inert, non-absorptive materials that supports the continuous culture of functional, bioprinted ICO-laden hepatic constructs. By enabling stable, long-term perfusion of these 3D-printed liver units, the system provides a controlled environment for drug toxicity testing. We demonstrated its utility by evaluating prolonged acetaminophen (APAP) exposure, revealing toxicity responses that are not captured in static cultures or Matrigel-based systems. The platform’s ability to enhance liver organoid maturation and detect progressive, low-level toxicity over extended culture periods of 7 days more closely reflects in vivo patterns of drug-induced liver injury. As such, this platform offers a robust tool for studying mechanisms of prolonged hepatotoxicity, improving compound screening, and advancing regenerative medicine approaches that require physiologically relevant liver models.

## Data Availability

The datasets used in this study are publicly available. The datasets can be found through the following link: https://dgk.yoda.uu.nl/research/browse?dir=%2Fresearch-neoliver (accessed on 15 January 2026).

## Supplementary Materials

The following supporting information can be downloaded at https://www.mdpi.com/article/10.3390/cells15151342/s1, Table S1. Basic information of donors; Table S2. List of primers; Table S3. List of antibodies; Figure S1. Setup of perfusion system; Figure S2. Brightfield images of ICOs pre- and post-bioprinting; Figure S3. HE staining of organoid-laden constructs before and after 5 days differentiation in static and perfused condition; Figure S4. Relative gene expression of constructs after 7 days APAP exposure under Matrigel culture, static and perfused conditions from two independent donors.
