# Supplementary material for: Rapid Volumetric Bioprinting Coupled with Dynamic Perfusion Enhances Human Hepatic Organoid Toxicity Testing
Source: Cells. 2026 Jul 27;15(15):1342. doi: 10.3390/cells15151342 (PMC13465539; doi:10.3390/cells15151342)
Supplement: Supplementary file 1 [file cells-15-01342-s001.zip › cells-4418576-supplementary.pdf]

Supplemental tables

Table S1. Basic information of donors

Donor name	gender	age
EU12	female	56
EU13	male	51
EU15	female	64

Table S2. List of primers

Gene	Forward primer	Reverse primer	Annealing Temp [°C]
RPS5	TGCAGGATTACATTGCAGTG	CATCATGGAGTTAGTGAGGC	60
RPL13A	GTGAAGGCATCAACATTTCTG	GATAGGCAAACTTTCTTGTAGG	60
ALB	GTTCGTTACACCAAGAAAGTACC	GACCACGGATAGATAGTCTTCTG	63.7
CYP3A4	TTTTGTCCTACCATAAGGGCTTT	CACAGGCTGTTGACCATCAT	62.5
MPR2	GGGATCTCTTCCACACTGGAT	CATACAGGCCCTGAAGAGGA	69
CYP1A2	ATCTTTGGAGCAGGATTTGAC	CTTCTGGATCTTCCTCTGTATCTC	63
CYP2C9	GACAGAGACGACAAGCAC	AATCACACGTTCAATCTCTTCC	61.6
BAAT	GAGGCTGCCAACTTTCTCCT	AGTACCGTGGCTGTGACTTG	60
TP53	TTGCAATAGGTGTGCGTCAGA	AGTGCAGGCCAACTTGTTCAG	60
CDKN1A	CCTCATCCCGTGTTCTCCTTT	GTACCACCCAGCGGACAAGT	60

Table S3. List of antibodies

Antigen	Source and cat. number	Host	Dilution	Incubation
E-cadherin	BD bioscience, 610181	mouse	1:100	O/N at 4°C
HNF4α	Novus Biologicals, NBP1-89679	rabbit	1:50	O/N at 4°C
MDR1	Novus bio, NBP1-90291	rabbit	1:200	O/N at 4°C
CK18	Santa Cruz, sc-51582	mouse	1:100	O/N at 4°C
Anti-mouse Alexa 488	Invitrogen, A-11029	goat	1:200	1h at RT
Anti-rabbit Alexa 568	Invitrogen, A-11036	goat	1:200	1h at RT

Supplemental figures

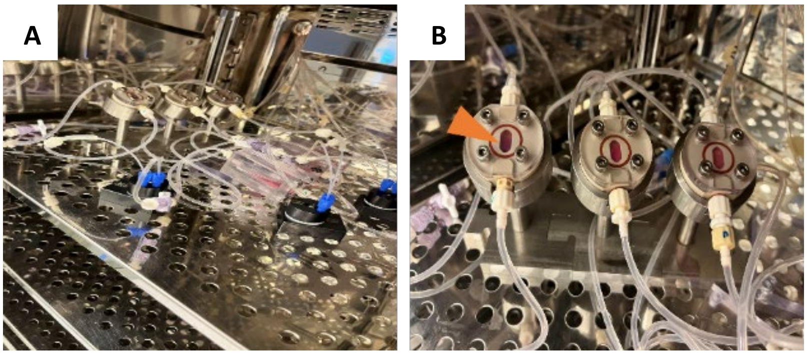

Figure S1. Setup of perfusion system. A) Perfusion aliments were connected by tubes and kept in a humified atmosphere of 95% air and 5% CO_2 at 37˚C. **B)** Three separate bioreactors were arranged in a modular system during the experiment, with constructs placed in each bioreactor (indicated by arrow).

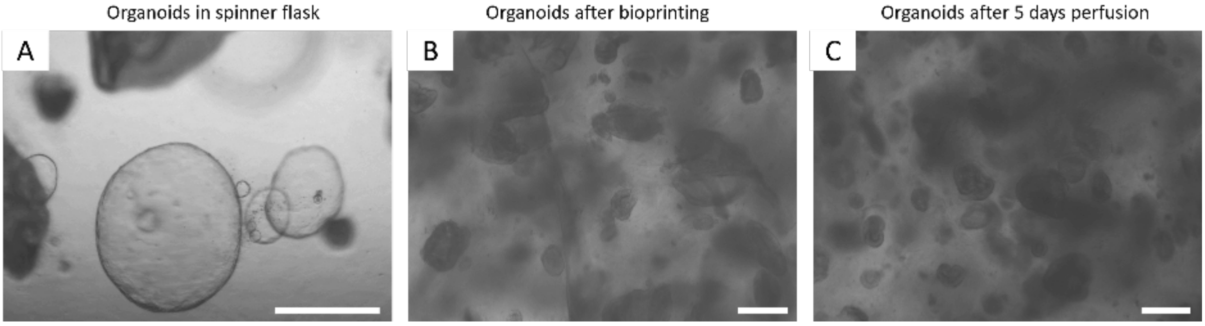

Figure S2. Brightfield images of ICOs pre- and post-bioprinting. A) ICOs were cultured in spinner flasks with EM supplemented with 10% vol/vol Matrigel to expand 45-76 million ICOs before volumetric bioprinting. **B)** Organoid-laden constructs were bioprinted with gelMA by VBP. **C)** Constructs were perfused in perfusion system for 5 days under DM. Scale bar=500 µm.

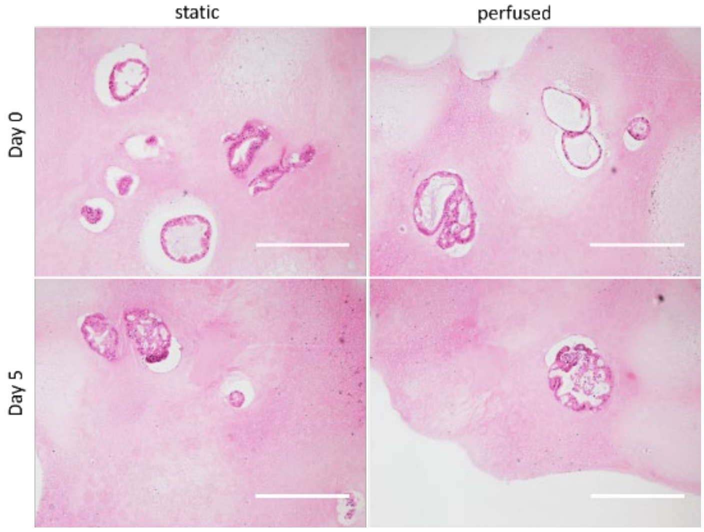

Figure S3. HE staining of organoid-laden constructs before and after 5 days differentiation in static and perfused condition. Bioprinted hepatic constructs were placed in bioreactor or T25 flasks with differentiation media. Three different donors were analyzed in independent experiments. HE staining

of paraffin-embedded constructs demonstrated visible ICOs after 5 days differentiation in static and perfused condition. Scale bar=200 µm.

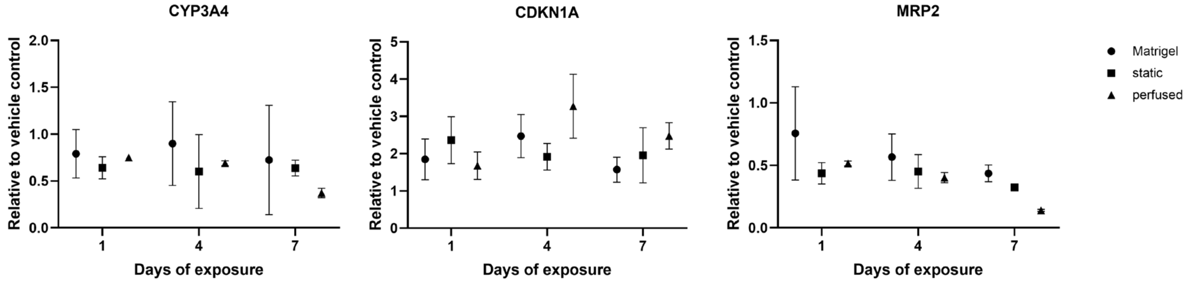

Figure S4. Relative gene expression of constructs after 7 days APAP exposure under Matrigel culture, static and perfused conditions from two independent donors (N=2 independent donors, n=3 technical replicates per donor)**.** Hepatocyte marker (CYP3A4) and and canalicular marker (MRP2) were determined by qRT-PCR and normalized to vehicle control. Cyclin-dependent kinase inhibitor 1A (CDKN1A) as a oxidative stress responses marker determined by qRT-PCR and normalized to vehicle control.
